# Interdisciplinary approach for the treatment of periodontally compromised malpositioned anterior teeth: a case report

**DOI:** 10.4076/1757-1626-2-8568

**Published:** 2009-07-20

**Authors:** Adrian Kasaj, Heiner Wehrbein, Aristea Gortan-Kasaj, Christoph Reichert, Brita Willershausen

**Affiliations:** 1 Department of Operative Dentistry and Periodontology, Johannes-Gutenberg UniversityMainzGermany; 2 Department of Orthodontics, Johannes-Gutenberg UniversityMainzGermany; 3 Department of Orthodontics, Rheinische Friedrich-Wilhelms-UniversityBonnGermany

## Abstract

Today many adult patients with periodontal disease demonstrate positioning of teeth that comprise their ability for proper mechanical tooth cleaning of approximal tooth surfaces. With adequate combined periodontal-orthodontic treatment it is possible to re-establish a healthy and well-functioning dentition. However, while orthodontic treatment can realign periodontally affected teeth, esthetic appearance may be compromised by gingival recession due to alveolar bone dehiscences or fenestrations in combination with a thin gingival biotype. This article reports an interdisciplinary (periodontic, orthodontic, restorative) approach for the treatment of a periodontally compromised patient with anterior dental malalignment. Periodontal therapy, including periodontal plastic surgery to obtain root coverage as well as orthodontic treatment by means of a miniscrew implant anchorage were used to achieve stable periodontal conditions and successful esthetic and functional final results.

## Introduction

Orthodontic tooth movement may provide a substantial benefit to periodontal therapy. Today many adult patients with periodontal disease exhibit problems with tooth malpositioning (e.g. frontal crowding) that comprise their ability for proper mechanical tooth cleaning of approximal tooth surfaces. The correction of malpositioned teeth permits the patient better access for oral hygiene and can improve the morphology of marginal soft and hard tissues. Adequate periodontal and orthodontic treatments have been shown to improve the periodontal condition and to re-establish a well-functioning dentition provided that an efficient plaque control is maintained [1]. Moreover, orthodontics gives the opportunity to improve the appearance of the esthetic zone. However, a different orthodontic treatment approach is required in periodontally compromised patients in terms of stabilizing anchorage system, force system, retention, as well as plaque control during treatment. More recently, great emphasis has been placed on the miniscrew type of temporary anchorage devices (TADs) becoming an accepted treatment option to more traditional methods of establishing orthodontic anchorage [2]. The TADs can provide a fixed anchorage for various tooth movements, are easily placed and removed, reduce overall treatment time requiring minimal patient compliance.

This clinical report describes an interdisciplinary (periodontic, orthodontic, restorative) approach for the treatment of a periodontally compromised patient with anterior dental crowding. Periodontal therapies, including periodontal plastic surgery as well as orthodontic treatment by means of a miniscrew implant anchorage were used to achieve stable periodontal conditions and successful esthetic and functional final results.

## Case presentation

**Figure 1.**
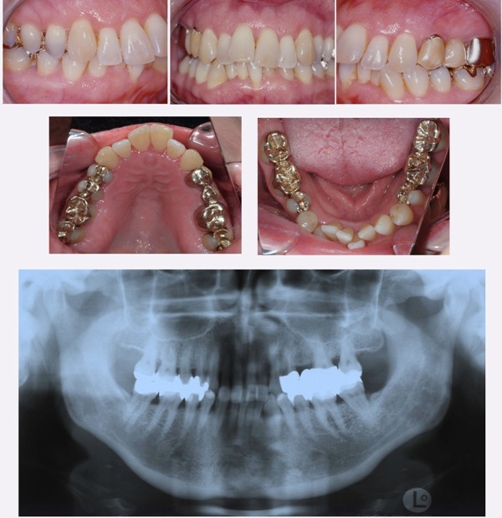
Pretreatment intraoral photographs and panoramic radiograph.

**Figure 2.**
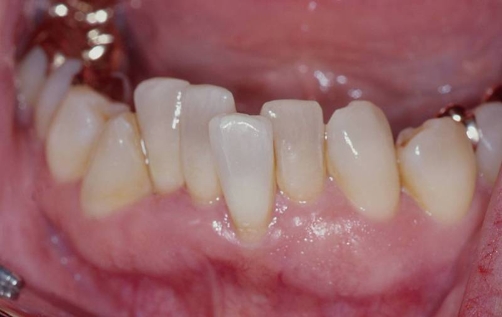
Initial clinical view of the anterior crowding in the lower anterior segment.

**Figure 3.**
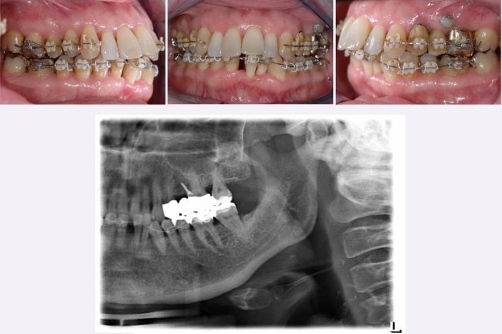
Orthodontic treatment included a miniscrew implant in region 25.

**Figure 4.**
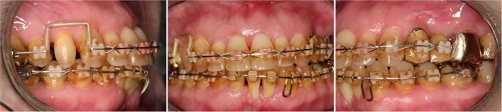
Final stage of orthodontic treatment after removal of the miniscrew implant.

**Figure 5.**
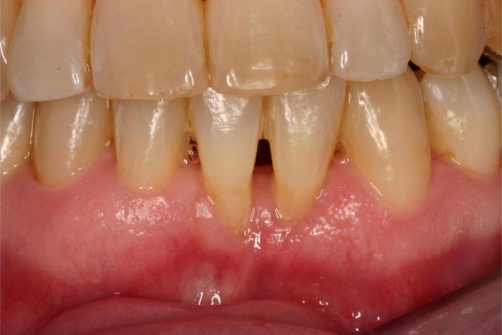
Clinical situation after orthodontic treatment showing a gingival recession in the esthetic area of teeth 32 and 41.

**Figure 6.**
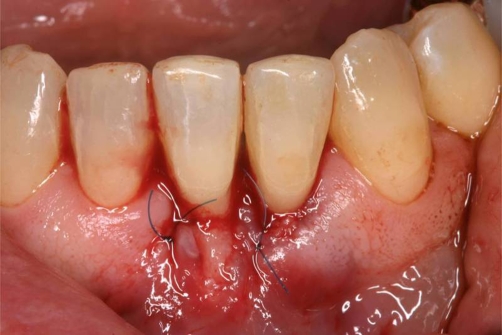
Placement of a connective tissue graft employing the “envelope” technique.

**Figure 7.**
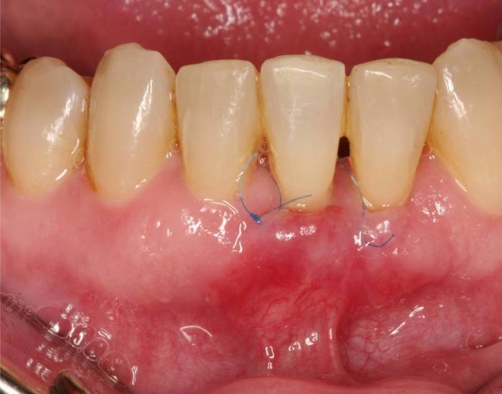
Clinical situation 10 days postoperative.

**Figure 8.**
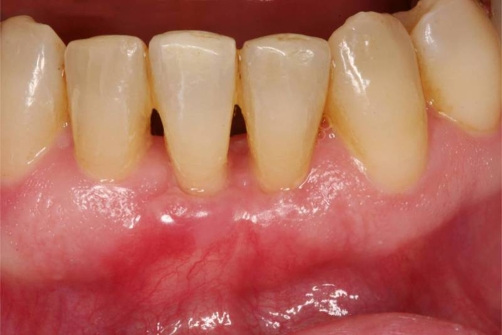
Clinical situation 3 months postoperative.

**Figure 9.**
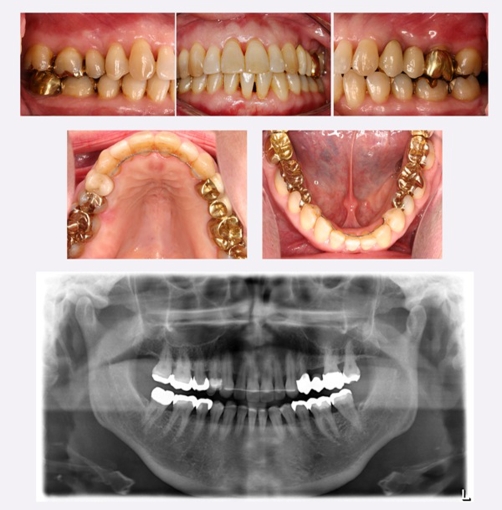
Postactive treatment intraoral photographs and panoramic radiograph.

A 61-year-old, German, Caucasian, systemically healthy, nonsmoking female presented with the chief complaint of bleeding gums and increasing frontal crowding of the mandibular and maxillary anterior teeth. The dental history showed that the patient had been receiving routine dental care at the University Clinic for several years. There was no history of periodontal treatment. A periodontal examination and charting were performed including assessment of probing depths (PDs), clinical attachment levels (CALs), full mouth bleeding (gingival bleeding index: GBI) and plaque scores (plaque control record: PCR). Generalized pocket depths ranging from 4 to 7 mm and gingival recession ≤2 mm were present throughout the dentition. The measurements for PDs and CALs were performed at six sites per tooth (mesio-buccal, mid-buccal, disto-buccal, disto-lingual, mid-lingual, and mesio-lingual). The occlusal examination revealed Angle Class I molar relationship bilaterally for the first molars and canines (Figure 1). The upper incisors showed pathologic anterior migration and rotations, whereas the lower anterior segment demonstrated anterior crowding with a severely protruted left central incisor (Figures 1, 2). Radiographic examination showed generalized, moderate, horizontal bone loss in both arches (Figure 1). Given the presented information, a diagnosis of moderate to advanced generalized chronic periodontitis with anterior dental crowding was made. Before starting orthodontic treatment, the patient received periodontal treatment. The treatment comprised oral hygiene instructions, supragingival scaling, and subgingival instrumentation using the Vector^TM^-ultrasonic system (Dürr Dental, Bittigheim-Bissingen, Germany). Following debridement, all periodontal pockets ≥5 mm received additional a biodegradable chlorhexidine chip (PerioChip®, Dexcel Pharma GmbH, Alzenau, Germany) for the controlled delivery of chlorhexidine. After periodontal treatment, the patient acquired good plaque control and clinically healthy gingiva. Probing depths were less than 4 mm with no signs of bleeding upon probing throughout the dentition and the patient was referred to the orthodontic department for further orthodontic management of malpositioned teeth in the mandibular and maxillary anterior area. Under local anesthesia, the mandibular left first incisor was extracted by minimal trauma to the gingival tissues in order to gain adequate space for the orthodontic treatment of the mandibular anterior crowding. To obtain a stationary anchorage for the distalization of the posterior teeth in the upper jaw a miniscrew implant was placed in the alveolar bone in region 25 (Figure 3). Multibracket appliance was used in the upper and lower jaw to improve the alignment of the mandibular and maxillary anterior teeth. The levelling and alignment were initiated with sectional wires and light forces. After 24 months of active orthodontic treatment acceptable overjet and overbite were achieved and the frontal crowding resolved (Figure 4). Three months before removal of the multibracket appliances, the miniscrew implant was removed. After the removal of the multibracket appliances, the maxillary and mandibular teeth were stabilized by bonded lingual canine-to-canine retainers. The patient was seen every 4 weeks for periodontal maintenance during the orthodontic treatment and home care was emphasized. During active orthodontic treatment and retention, PDs and CALs were maintained at the levels achieved after periodontal treatment. After 6 months of retention, a correction of recession of the gingival margin in the esthetic area 41 (Miller Class III) was performed with the placement of a connective tissue graft employing the “envelope” technique [3] (Figures 5-8). Briefly, a connective tissue graft was harvested from the palatal area between the canine and the first molar according to the “trap door” approach and the exposed root surface at the recipient site was planed with curettes and burs. The connective tissue was transplanted into a supraperiosteal envelope-like pouch prepared at the recipient site and stabilized with two sutures using non-resorbable #5-0 suturing material (Premilene®, Braun Aesculap, Tuttlingen, Germany). The pouch provides a dual blood supply to the graft from the superior and inferior connective tissue surfaces in contact with the graft. The postoperative healing was uneventful and sutures were removed after 1 week. With this technique, substantially improved root coverage with increased width and thickness of keratinised gingival tissue was obtained. The final restorative treatment included a new fixed metal ceramic bridge replacing the maxillary left second premolar, insertion of a gold inlay on the maxillary right first premolar and a new full coverage gold crown on tooth 47 (Figure 9).

## Discussion

Periodontal disease can lead to pathologic migration of involved teeth and cause severe functional and esthetic problems. The clinical manifestations of pathologic migration, such as rotation, elongation and spacing/crowding of the incisors have been found in 30% to 50% of patients with moderate to severe periodontal disease [4,5].

In the present case report pathologic migration of maxillary/mandibular incisors, crowding of the mandibular incisors and periodontal disease were significant. The migration of the maxillary incisors, as well as the crowding of the mandibular incisors was believed to be the result of pathological migration, since the patient reported no previous crowding. Although negative influences of tooth malposition on the periodontal tissues are still questioned [6], several studies demonstrated the interrelationship between crowding of frontal teeth and periodontal disease [7-9]. The comparison of the microbial composition in the subgingival plaque of adult crowded versus non-crowded dental regions demonstrated to have more plaque accumulated in crowded areas with more species of periodontopathogens in comparison to non-crowded areas [10]. In this case the combined periodontal and orthodontic treatment resulted in stable periodontal health exhibiting probing depths less than 4 mm with no signs of bleeding throughout the dentition. In addition, eliminating mandibular anterior crowding helped to improve bone support and secure access for plaque control. However, although it has been demonstrated that orthodontic tooth movement is no more a contraindication in the therapy of adult patients affected by severe periodontal disease [11], it should be carefully performed. Thus, lighter orthodontic force systems should be applied to periodontally compromised teeth because they can move easily, and greater orthodontic forces can negatively affect the periodontal membrane [12]. In order to obtain appropriate orthodontic anchorage in the upper left posterior segment a titanium screw was used in the present case. These devices have demonstrated high efficiency in clinical application and represent a viable alternative to achieve sufficient anchorage against orthodontic forces [2].

It has to be emphasized that the key element in the orthodontic management of adult patients with periodontal disease is to eliminate plaque accumulation and gingival inflammation. In contrast, the lack of oral hygiene instructions and periodical check-ups throughout orthodontic treatment results in bone resorption [13]. In the present case, initial periodontal conditions were improved by scaling and root planning in conjunction with the controlled subgingival delivery of chlorhexidine before orthodontic treatment. During orthodontic treatment, a strict oral hygiene program was applied, including oral hygiene control and professional tooth cleaning every 4 weeks. However, after orthodontic treatment a marked loss of the interdental bone and soft tissue height between the mandibular incisors as well as a labial recession on the first mandibular incisor compromised the esthetic result. The interdental papilla loss can be contributed to the fact that the contact point shifted too far incisally on the triangular crowns that have not had a normal interdental wear pattern. A further contributing factor could be the destruction of the crestal bone between the incisors. Today we know that the presence of papilla strictly depends on the distance between the contact point and the crest of the bone [14]. A method of correcting this problem is to recontour the mesiodistal surfaces of the incisors by moving the roots of the teeth together, which will result in lengthening and moving the contact point apically. Another treatment option is the use of resin-based composites to close the interdental space of adjacent teeth. These treatment options were discussed with the patient. However, in this case the patient’s chief complaint after orthodontic therapy was the labial recession on the first mandibular incisor which was successfully treated by placement of a connective tissue graft employing the “envelope” technique [3]. The labial recession may be caused by facial tooth movement out of the alveolar bone in combination with a thin soft tissue biotype [15].

The planning of retention and the stability of orthodontic treatment requires greater consideration in periodontally compromised patients. Thus, permanent retention is often part of the total treatment plan for these patients. A long-term lingual-bonded wire retention was applied in the upper and lower arch.

In conclusion, the interdisciplinary treatment approach that involved nonsurgical periodontal therapy, orthodontic tooth movement with the use of a miniscrew implant for skeletal anchorage, periodontal plastic surgery, and final restorative treatment resulted in significant functional, esthetic and periodontal improvements.

## Abbreviations

CALs: clinical attachment levels
GBI: gingival bleeding index
PCR: plaque control record
PDs: probing depths
TADs: temporary anchorage devices
